# Looking for the Genes Related to Lung Cancer From Nasal Epithelial Cells by Network and Pathway Analysis

**DOI:** 10.3389/fgene.2022.942864

**Published:** 2022-07-18

**Authors:** Noman Qureshi, Jincheng Chi, Yanan Qian, Qianwen Huang, Shaoyin Duan

**Affiliations:** Department of Medical Imaging, Zhongshan Hospital, School of Medicine, Xiamen University, Xiamen, China

**Keywords:** nasal epithelium, lung cancer, WGCNA, modules, hub gene

## Abstract

Previous studies have indicated that the airway epithelia of lung cancer-associated injury can extend to the nose and it was associated with abnormal gene expression. The aim of this study was to find the possible lung cancer-related genes from the nasal epithelium as bio-markers for lung cancer detection. WGCNA was performed to calculate the module–trait correlations of lung cancer based on the public microarray dataset, and their data were processed by statistics of RMA and *t*-test. Four specific modules associated with clinical features of lung cancer were constructed, including blue, brown, yellow, and light blue. Of which blue or brown module showed strong connection to genetic connectivity. From the brown module, it was found that HCK, NCF1, TLR8, EMR3, CSF2RB, and DYSF are the hub genes, and from the blue module, it was found that SPEF2, ANKFN1, HYDIN, DNAH5, C12orf55, and CCDC113 are the pivotal genes corresponding to the grade. These genes can be taken as the bio-markers to develop a noninvasive method of diagnosing early lung cancer.

## Introduction

In recent 50 years, the morbidity and mortality of lung cancer have significantly increased, and the 5-year mortality rate is up to 80%. The main cause is lack of effective diagnostic tools to detect early lung cancer (Pisani et al., 1999). Although high-resolution CT (HRCT) and bronchoscopy increases the diagnostic sensitivity, the screening is not feasible because of high cost or complex operation (Gupta et al., 2009; Cannioto et al., 2018; Asaad Zebari and Emin Tenekeci, 2022). Despite low complications, bronchoscopy cannot identify the extent of cancer or the size and location of small or peripheral lung cancers (Khan et al., 2016). Previous studies have shown that some gene expression of epithelial cells in the entire bronchial airway is significantly different between normal people and smokers with lung cancer and proved that the existence of some pivotal genes in the nasal epithelium was closely related to lung cancer. These genes have been applied as biomarkers and classifiers to identify the lung cancers from benign diseases (Khan et al., 2016; Team, 2017). It was suggested that this analysis is an additional noninvasive and convenient detection approach for lung cancer.

The latest progress in gene interaction network methodology is to study the potential internal relationship between functional gene clusters and clinical features (Sun et al., 2017; Timmins and Ashlock, 2017). Identifying important modules related to clinical features is helpful to infer the tumor mechanism and establish new targets for diagnosis or therapy. Weighted gene co-expression network analysis (WGCNA) is an effective approach based on “guilt-by-association”. It is used for identifying gene modules as candidates for biomarkers. WGCNA creates in terms of large-scale gene expression reports and the identification of centrally sited genes or hub genes, which drive key cellular signaling pathways. The systematical biology method has been used to identify the hub genes in high-grade osteosarcoma and small cell lung cancer (SCLC) and to find potential therapeutic targets (Ning et al., 2016; Shakeel et al., 2020). This study was planned to make improvements in biology methods, which might increase the diagnostic efficiency of lung cancer at early stages, with low price and non-trauma.

## Materials and Methods

### Data Filtering

The expressional profile of GSE80796 was installed from the Gene Expression Omnibus (GEO) (http://www.ncbi.nlm.nih.gov/geo/). The data and clinical traits were reserved to analyze the difference in gene expression between the nasal epithelia of patients with less than 3 cm of early lung cancer and benign pulmonary nodule in different genders (6). Finally, there were a total of 197 samples, including 100 samples of benign pulmonary nodule (62 cases with tuberculoma, 23 with inflammatory pseudotumor, 9 with sclerosing hemangioma, and 6 with hamartoma) and 97 cases of early lung cancer (81 NSCLC and 16 SCLC).

### Data Preprocessing and Identification of Genes

At first, chip data were downloaded, including the background correction, normalization preprocess, and calculation of gene expression values. Robust multi-array average (RMA) and R language (McCall et al., 2010) were applied in the affy package, and the ComBat method was used in adjustment for batch effects. Subsequently, differentially expressed genes (DEGs) of nasal epithelia between early lung cancer and benign pulmonary nodule were identified using *t*-test in the linear models for microarray data, and the top 3,600 DEGs in the order of |logFC| were chosen for the construction of WGCNA (Langfelder and Horvath, 2008).

### Construction of a Clustering Tree for WGCNA

The WGCNA package in R language was used to construct the gene co-expression network analysis of nasal epithelia gene expression for both male and female and then continually to compare and screen the consensus modules of nasal epithelia gene expression in different genders.

### Brief Process

The process contained the following steps: 1) created a correlation matrix of the pairs of genes from all samples. 2) Chose the proper soft threshold. 3) With the proper power value, performed the automatic network construction and module detection with the major parameters: max BlockSize of 5,000, min ModuleSize of 40, deep Split of 4, and merge CutHeight of 0.25. 4) Built a hierarchical clustering dendrogram of gene expression data for each dataset and identified the shared functional modules.

### Calculation of the Correlation and Hub Gene Identification

In order to determine the correlation between gene expression modules and clinical traits, the age and smoking history (smoking time, pack/years) of patients with lung cancer were chosen and analyzed. As for the hub genes, Cytoscape software was used for constructing the scale-free WGCNA for selected modules (Shannon et al., 2003). The cytoHubba package from Cytoscape was performed to extract the top 20 hub genes selected by 12 different algorithms, and mutual hub genes were then chosen by comparison of the top 20 hub genes. In order to select gene modules, the pathway functional enrichment analyses, including the Gene Ontology (GO) and Kyoto Encyclopedia of Genes and Genomes (KEGG), were performed by the Database for Annotation, Visualization and Integrated Discovery (DAVID). These gene functions were analyzed at the molecular level.

## Results

### Screening of DEGs

Top 3,600 DEGs in the order of |logFC| were identified in the samples of early lung cancer by comparing with those of benign pulmonary nodule; there were 1,745 upregulated genes, and 1,855 downregulated genes.

### Construction of Co-Expression Module of Lung Cancer

**FIGURE 1:**
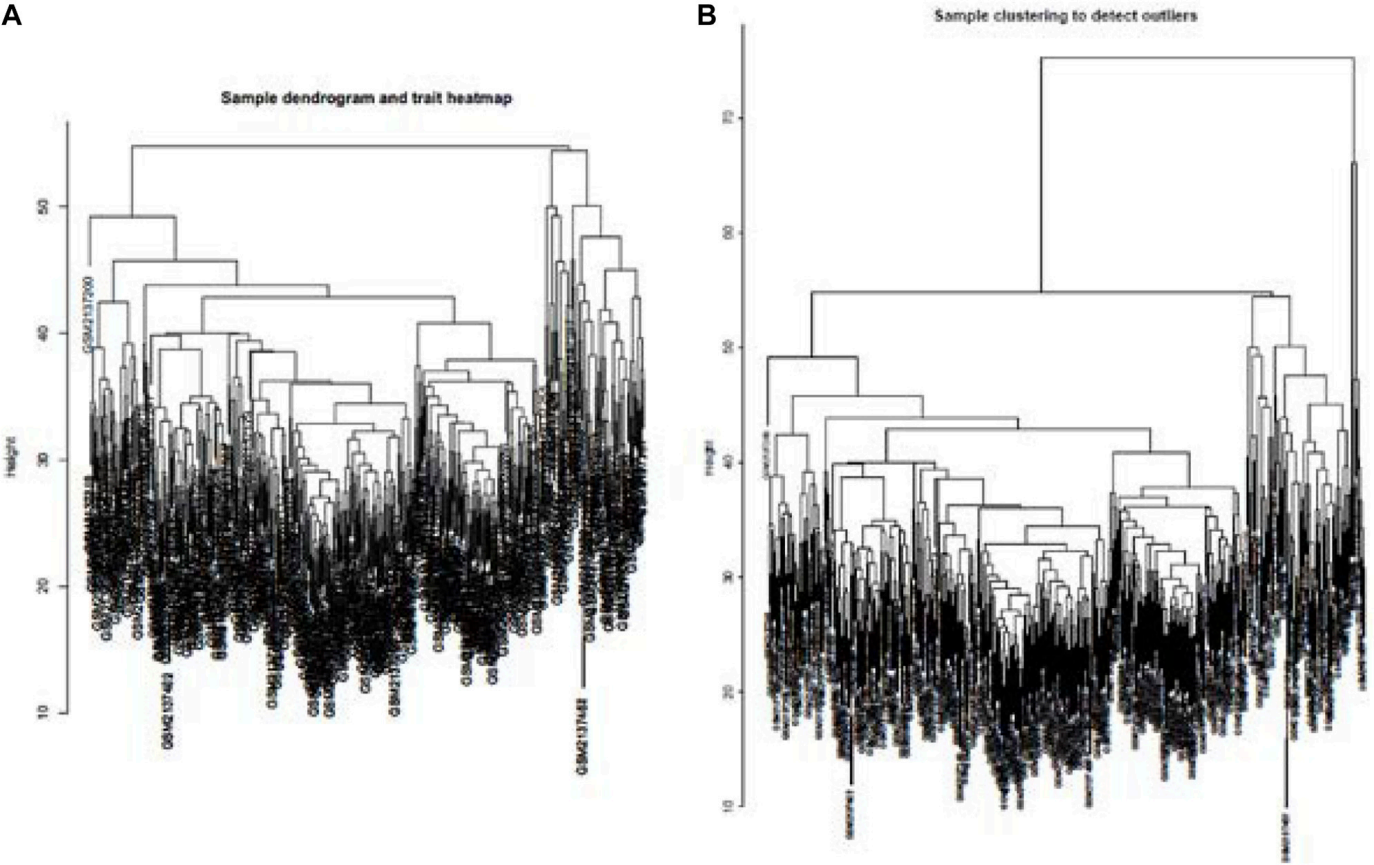
lustering all the samples in the input sets **(A)**. Height of data >55 cannot be well clustered with other data. Therefore, as outliers, data with height >55 were removed. Dendrogram of residual samples was constructed as shown **(B)**.

**FIGURE 2:**
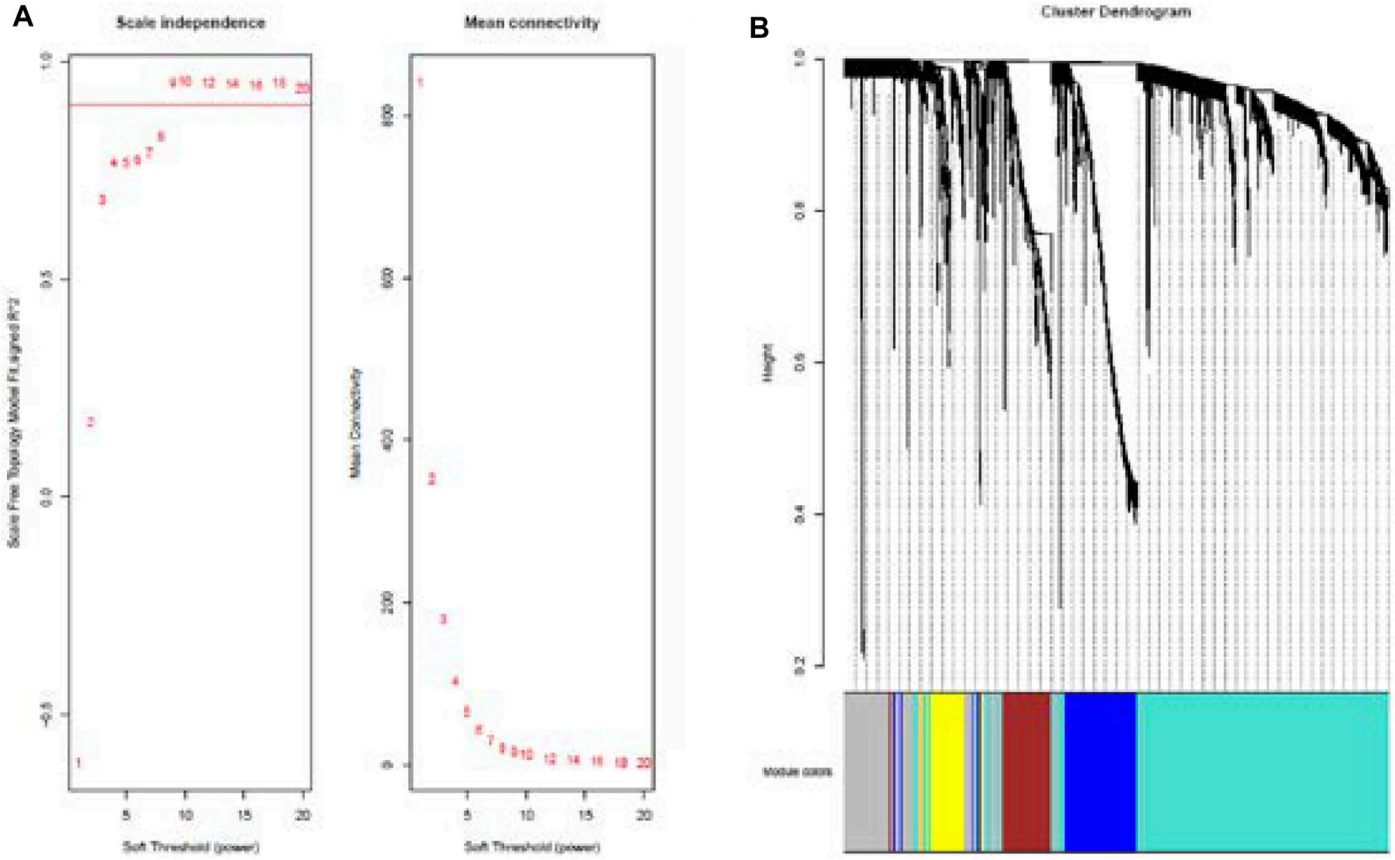
Analysis of network topology for various soft-thresholding powers **(A)**. Summary network indices (*y*-axes) as functions of the soft-thresholding power (*x*-axes). Numbers in the plots indicate the corresponding soft-thresholding powers. The plots indicate that approximate scale-free topology is attained around the soft-thresholding power of 9 for the sets. Because the summary connectivity measures decline steeply with increasing soft-thresholding power, it is advantageous to choose the lowest power that satisfies the approximate scale-free topology criterion. Cluster dendrogram and co-expression network modules for differentially expressed genes in the nasal epithelium of lung cancer and benign pulmonary nodule **(B)**. Gene dendrogram obtained by clustering the dissimilarity based on consensus topological overlap with the corresponding module colors indicated by the color row.

**FIGURE 3:**
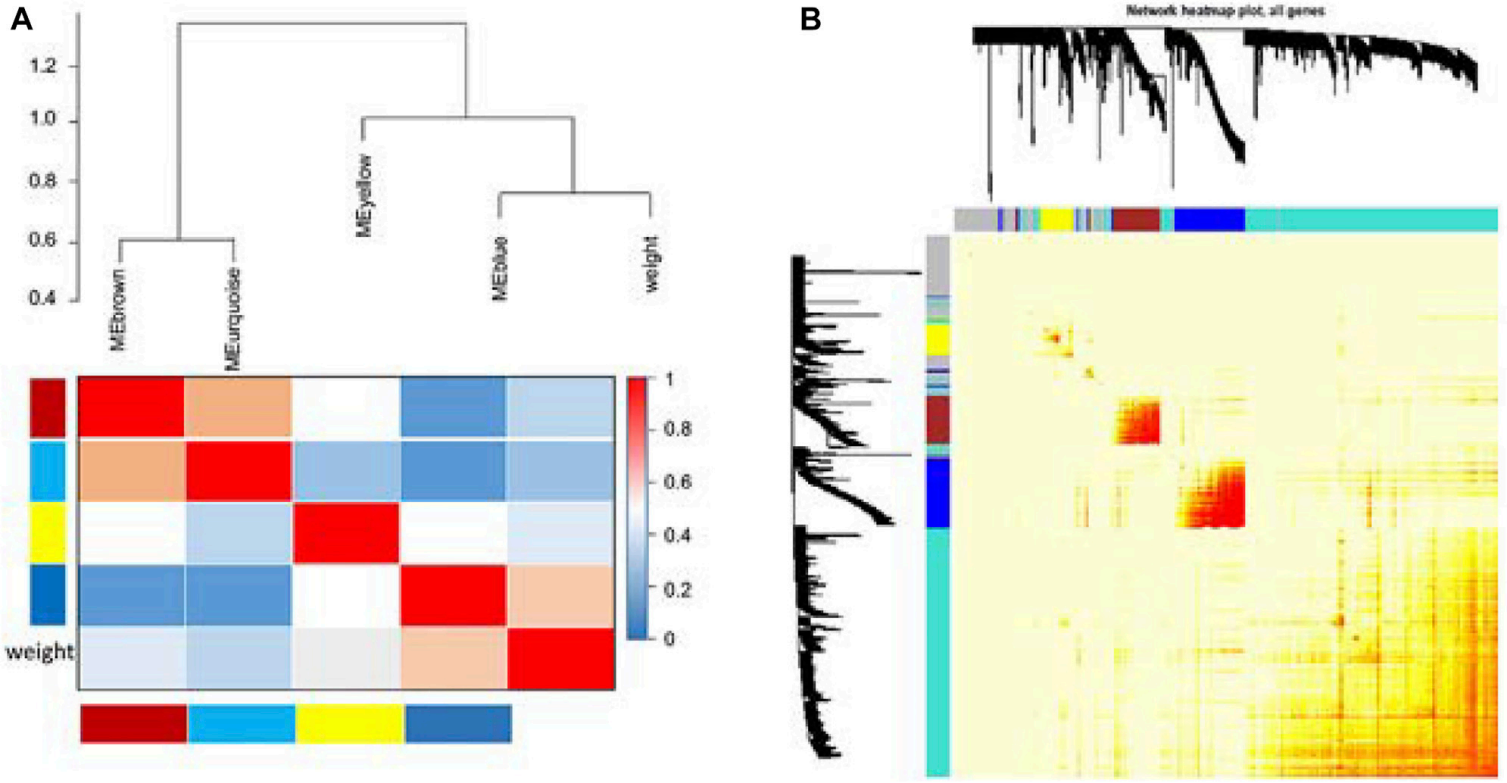
Comparisons between four modules. Genes in the blue module have the highest correlation **(A)**. Average linkage hierarchical clustering with the topological overlap dissimilarity measure was used to identify gene co-expression modules. The gene network was visualized using a heatmap plot. Light color represents low overlap, while the progressively darker red color represents higher overlap. Blocks of darker colors along the diagonal are the modules. Gene dendrogram and module assignment are also shown along the left side and at the top **(B)**.

Cluster analysis of DEGs is clearly shown in Figure 1A. Those samples were cut whose expression level was higher than 50 (Figure 1B). The soft threshold is the most important parameter. First, the soft threshold was selected (Figure 2A). When the power value was equal to 9, the degree of independence was up to 0.9, and the average connectivity was high. Five different gene co-expression modules were identified and displayed in different colors (Figure 2B). The gray module contained all the modules that could not be allocated to other modules, and the interaction of the co-expression modules showed that the thermograph depicted the topological overlap matrix of all genes. By constructing the TOM, these genes in the blue module had the highest correlation (Figures 3A,B).

### Analysis of WGCNA Network

**FIGURE 4:**
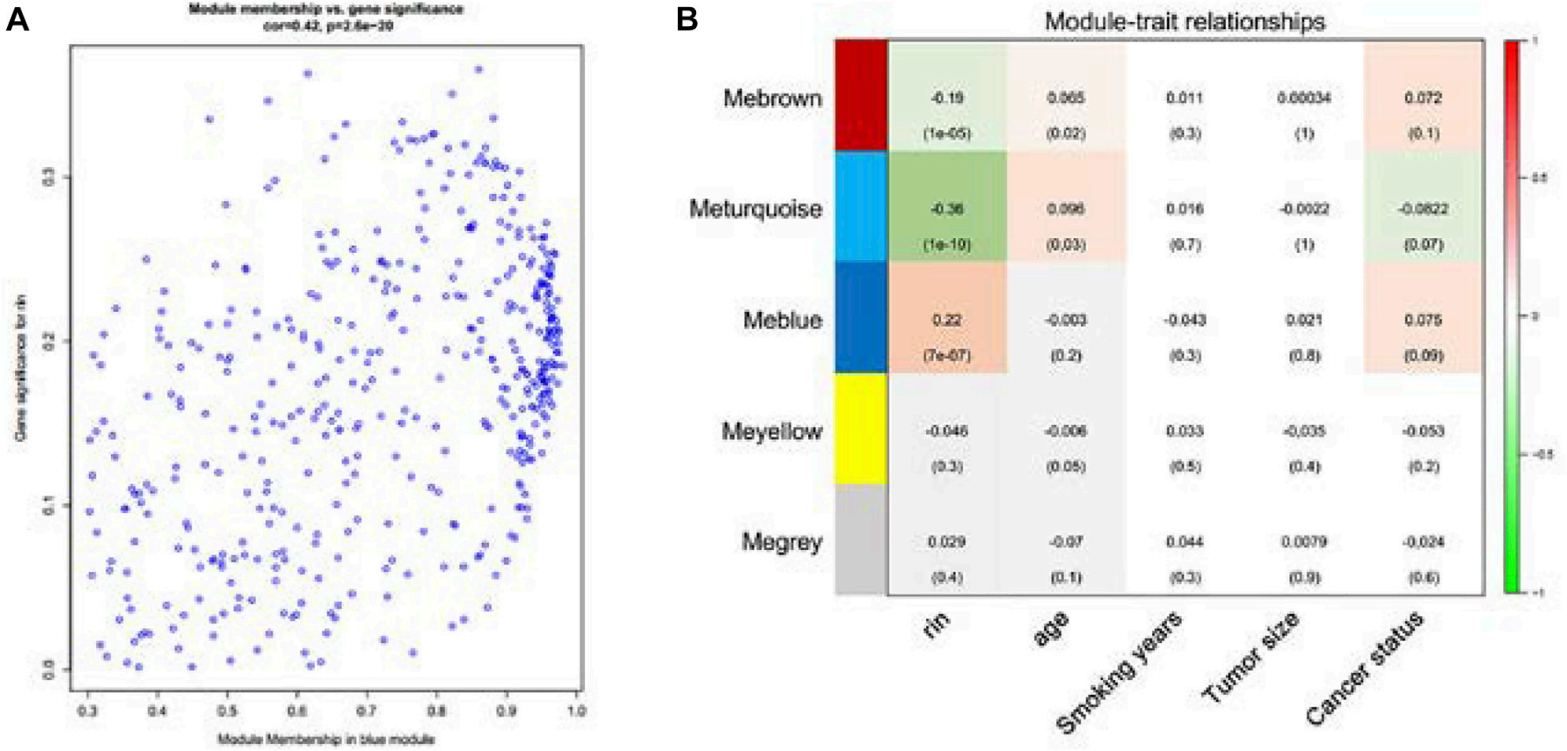
Scatterplot of gene significance (GS) for lung cancer status vs. module membership (MM) in the blue module **(A)**. There is a highly significant correlation between GS and MM in the blue module. Correlation of gene co-expression modules with clinical traits in the training cohort (*n* = 196). Blue module is strongly correlated with early lung cancer, so the blue module was chosen to be further analyzed **(B)**.

Consensus relationships of consensus module eigengenes and clinical traits were presented as weak mutual correlations (*p* > 0.05), while the consensus module eigengenes and clinical traits showed significant correlations (*p* < 0.05) in the male and female data, respectively, which verified the conclusion of heterogeneity related with gender. There were scatter plots of GS and MM of blue and brown module genes, which had the highest correlation in the blue module (Figure 4A). The module feature relationship is displayed in Figure 4B. Their clinical features included age, smoking years, tumor size, and lung cancer status. Cluster analysis showed that the blue module was significantly correlated with the clinical characteristics of lung cancer.

### Gene Co-Expression and Hub Genes

**FIGURE 5:**
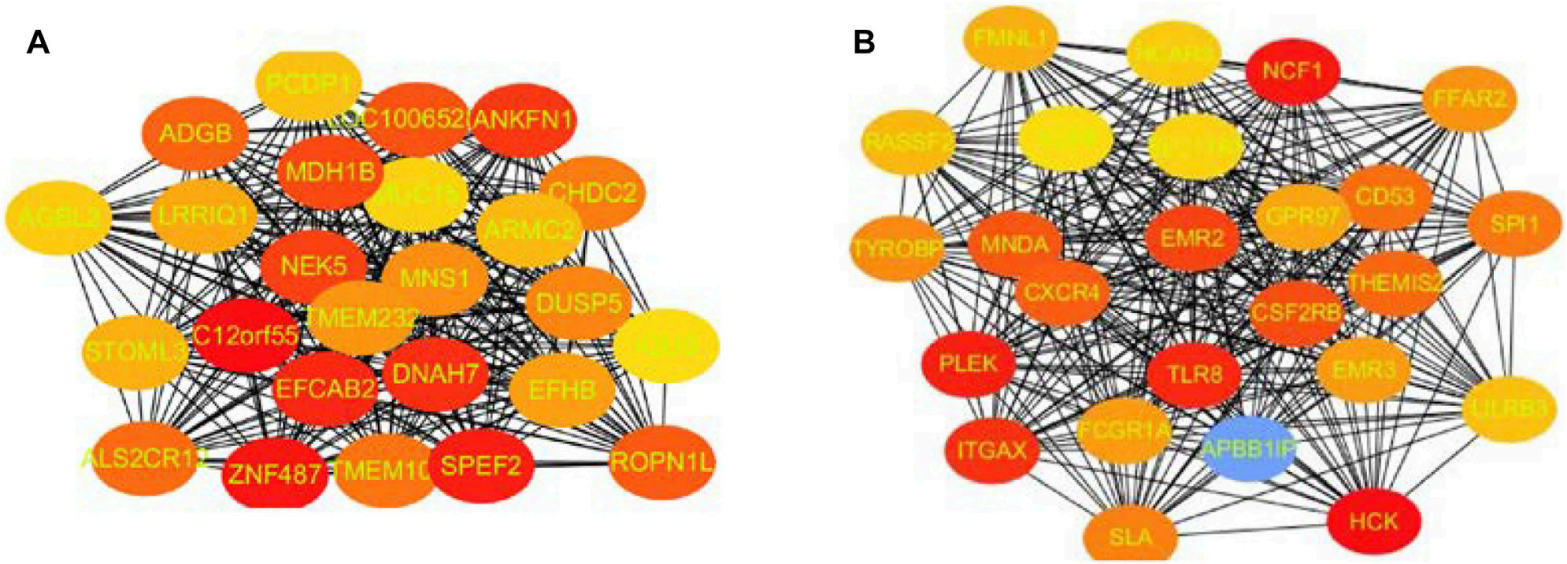
Top 25 hub genes calculated by MCC algorithms have strong correlations with early lung cancer. Progressively darker red color represents higher relationship in the blue module. C12orf55, ZNF487, EFCAB2, and ANKFN1 are the most closely related hub genes **(A)**. The hub genes were identified by MCC in the brown module. NCF1, PLEK, TLR8, and HCK are the most closely related hub genes in the brown module **(B)**.

In these genes, four specific modules of lung cancer were constructed as blue, brown, yellow, and light blue modules, and the blue and brown modules were strongly linked to genetic connectivity. Twelve algorithms of the cytoHubba package were used to calculate the hub genes and their connectivity in Cytoscape software. In the brown module, the hub genes identified were TLR8, HCK, NCF1, EMR3, CSF2RB, and DYSF; in the blue module, the pivotal genes identified were HYDIN, SPEF2, ANKFN1, DNAH5, C12orf55, and CCDC113 (Tables 1, 2). In every network, the color depth is directly proportional to its connectivity. Four specific modules associated with clinical features of lung cancer were constructed, including blue, brown, yellow, and light blue, of which blue or brown module showed strong connection to genetic connectivity (Figures 5A,B). TLR8, HCK, NCF1, EMR3, CSF2RB, and DYSF were the hub genes identified from the brown module, and HYDIN, SPEF2, ANKFN1, DNAH5, C12orf55, and CCDC113 were the pivotal genes identified from the blue module.

**TABLE 1:** Top 25 hub genes of the blue module through dataset 1.

P2	MCC3	DMNC4	MNC5	Degree	EPC6	BottleNeck
1	C12orf55	IL36G	—	—	—	DNAH6
2	ZNF487	KRT13	DNAH6	DNAH6	DNAH6	—
3	SPEF2	ACSS3	HYDIN	HYDIN	HYDIN	MAP3K19
4	EFCAB2	HSPB8	DNAH12	DNAH12	DNAH12	DNAH7
5	DNAH7	SPRR1B	C12orf55	C12orf55	C12orf55	APOBEC4
6	ANKFN1	CYP2G2P	LOC100652824	LOC100652824	LOC100652824	CCDC113
7	NEK5	ABCB11	DYNC2H1	DYNC2H1	ULK4	WDR49
8	MDH1B	RNU6-646P	ULK4	ULK4	DYNC2H1	HYDIN
9	LOC100652824	CEACAM6	SPEF2	SPEF2	NEK5	RUVBL1
10	ROPN1L	DCAKD	DNAH7	EFCAB1	EFCAB1	C7orf63
11	ADGB	SYTL5	ADGB	NEK5	SPEF2	SNORD116-1
12	ALS2CR12	HTR3A	WDR49	DNAH7	AK9	SNORD116-29
13	TMEM107	RNU2-50P	NEK5	WDR49	ATXN7L1	IFT88
14	CHDC2	CPA4	EFCAB1	ADGB	MNS1	C12orf55
15	DUSP5	RNU6-490P	WDR96	WDR96	RSPH4A	SNORD116-24
16	MNS1	DSG3	IQUB	MNS1	CC2D2A	TMEM231
17	TMEM232	KCNJ16	STK33	CASC1	MAP3K19	ARMC2
18	EFHB	PDLIM2	CCDC30	WDR65	TCTEX1D1	SNORA20
19	LRRIQ1	CCDC34	WDR65	CCDC30	WDR96	SNORAD116-15
20	STOML3	FABP5	CASC1	IQUB	IQUB	SNORAD115-32
21	ARMC2	SOX2	MNS1	ANKFN1	WDR65	IQCK
22	PCDP1	RNU6-955P	SPAG17	ATXN7L1	ANKFN1	DNAH2
23	AGBL2	CNTNAP3B	ANKFN1	EFCAB2	NEK10	NEK5
24	MUC15	—	CCDC113	WDR63	DNAH7	TCTEX1D1
25	IQUB	KRT6B	NEK10	CCDC39	WDR49	NME5

P	EcCentricity	Closeness	Radiality	Node_name	Stress	CC7
1	SNORD116-29	—	—	—	—	IL36G
2	SCARNA7	DNAH6	DNAH6	DNAH6	DNAH6	KRT13
3	SNORA20	HYDIN	HYDIN	HYDIN	HYDIN	ACSS3
4	SNORD116-15	DNAH12	DNAH12	DNAH12	DNAH12	DSG3
5	SNORD116-24	C12orf55	C12orf55	SNORD116-24	C12orf55	HSPB8
6	SNORD116-5	LOC100652824	LOC100652824	CCDC113	CCDC113	CEACAM6
7	SNORD116-25	DYNC2H1	DYNC2H1	DYNC2H1	ARMC2	SPRR1B
8	SNORD116-1	ULK4	ULK4	C12orf55	DYNC2H1	SYTL5
9	SNORD116-26	SPEF2	SPEF2	ARMC2	RPGRIP1L	RNU6-646P
10	RUVBL1	DNAH7	DNAH7	LOC100652824	LOC100652824	ABCB11
11	DNAH7	ADGB	WDR49	SNORD116-29	SNORD116-24	CYP2G2P
12	C14orf142	WDR49	NEK5	SNORA20	TCTEX1D1	HTR3A
13	CCDC113	NEK5	ADGB	SCARNA7	WDR65	SNORA28
14	NUCB2	WDR96	WDR96	WDR65	SNORA20	DCAKD
15	TCTEX1D1	EFCAB1	STK33	RPGRIP1L	SNORD116-29	RNU6-490P
16	ZMAT1	STK33	IQUB	ULK4	ZBBX	HPX
17	DTHD1	IQUB	EFCAB1	TCTEX1D1	SPATA18	KRT6B
18	FANK1	WDR65	WDR65	MAATS1	SCARNA7	SOX2
19	CCDC60	CCDC30	CCDC113	TMEM232	MAATS1	AZGP1
20	PACRG	CASC1	CASC1	ZBBX	ULK4	KCNJ16
21	SPEF2	MNS1	CCDC30	C9orf116	SNORD116-14	RCBTB1
22	VWA3A	SPAG17	DTHD1	DTHD1	C9orf116	KRT24
23	KIAA1377	ANKFN1	ANKFN1	SNORD116-5	SNORD116-1	ADIRF
24	ARMC2	CCDC113	ARMC2	ADGB	SNORD116-15	SNORD116-29
25	KIAA1841	DTHD1	MNS1	SPATA18	ADGB	LOC100131860

Notes: The hub gene was calculated by cytoHubba 1; parameters 2; maximal clique centrality 3; density of maximum neighborhood component 4; maximum neighborhood component 5; edge percolated component 6; clustering coefficient 7.

**TABLE 2:** Top 25 hub genes of the brown module through dataset 1.

P2	MCC3	DMNC4	MNC5	Degree	EPC6	BottleNeck
1	HCK	TPD52L2	—	—	—	—
2	NCF1	FCGR3A	HCK	FCGR3A	FCGR3A	DYSF
3	PLEK	NCF2	NCF1	NCF1	HCK	LILRB2
4	TLR8	—	FCGR3A	HCK	NCF1	SLED1
5	ITGAX	MIR23A	CSF2RB	GLT1D1	CSF2RB	PREX1
6	APBB1IP	NFIL3	GLT1D1	CSF2RB	PLEK	TAGAP
7	EMR2	CD14	EMR2	EMR2	FCGR1A	LOC254896
8	CSF2RB	FCGR1A	FPR1	FCGR1A	FPR1	EMR3
9	MNDA	MIR223	TAGAP	TAGAP	GLT1D1	SH2D3C
10	CXCR4	ITGAX	EMR3	EMR3	MNDA	—
11	THEMIS2	PLEK	MNDA	FPR1	TAGAP	FOSB
12	CD53	NFE2	GPR97	GPR97	LCP2	ZFP36
13	SPI1	SIRPB1	CSF3R	MNDA	EMR3	NFAM1
14	SLA	LINC00921	DYSF	LCP2	THEMIS2	PPP1R18
15	TYROBP	THEMIS2	TLR8	PLEK	ITGAX	PTPRC
16	FFAR2	P2RY13	APBB1IP	CSF3R	DYSF	CD14
17	EMR3	EVI2B	LCP2	DYSF	EMR2	TRIB1
18	FCGR1A	ARRB2	PLEK	BCL2A1	BCL2A1	IL1B
19	GPR97	TREM1	SLA	ITGAX	CSF3R	MNDA
20	FMNL1	TNFAIP6	FCGR1A	SLA	FCGR2A	FMNL1
21	RASSF2	PLXNC1	ITGAX	TLR8	SLA	RGS2
22	LILRB3	CHST11	LILRB3	FCGR2A	TLR8	LPCAT1
23	HCAR3	SELPLG	BCL2A1	APBB1IP	TREM1	FYB
24	SLC11A1	NABP1	RASSF2	LILRB3	LILRB3	FFAR2
25	AQP9	SELL	FCGR2A	RASSF2	RASSF2	CHSY1

P	EcCentricity	Closeness	Radiality	Node_name	Stress	CC7
1	CSRNP1	—	—	—	—	TPD52L2
2	HRH2	HCK	HCK	HCK	HCK	GZMB
3	OSM	NCF1	NCF1	SH2D3C	SH2D3C	HEY2
4	CLEC4E	FCGR2A	FCGR2A	NCF1	NCF1	MIR23A
5	ARID5A	CSF2RB	CSF2RB	CSF2RB	EMR2	LINC00921
6	CASS4	GLT1D1	EMR2	EMR2	EMR3	CD14
7	PLEKH02	EMR2	GLT1D1	EMR3	FCGR3A	NFIL3
8	LILRB3	FPR1	EMR3	LILRB2	CSF2RB	ARRB2
9	—	EMR3	FPR1	DYSF	ADAM8	RAB24
10	TLR8	MNDA	MNDA	GLT1D1	DYSF	NFE2
11	—	TAGAP	DYSF	ADAM8	GLT1D1	NCF2
12	SH2D3C	GPR97	TAGAP	CSF3R	CSF3R	RN7SKP78
13	PHOSPHO1	CSF3R	LCP2	TLR8	TLR8	GMIP
14	—	DYSF	CSF3R	TAGAP	LCP2	MIR223
15	CEBPD	LCP2	ITGAX	LCP2	SLA	EDN1
16	TPD52L2	TLR8	LILRB3	APBB1IP	LILRB2	CHST11
17	RAB24	ITGAX	GPR97	FCGR3A	WAS	P2RY13
18	RN7SKP78	PLEK	TLR8	WAS	TAGAP	NABP1
19	EGR2	FCGR1A	PLEK	SLA	ITGAX	SIRPB1
20	CNN2	SLA	FCGR1A	FPR1	—	ZC3H12A
21	FOSB	LILRB3	SLA	GPR97	FPR1	TNFAIP6
22	RCSD1	APBB1IP	APBB1IP	LILRB3	CYTH4	AOAH
23	TNFAIP6	BCL2A1	PROK2	DGAT2	APBB1IP	EVI2B
24	DYSF	PROK2	RASSF2	ALOX5	MOB3A	PRKCB
25	LILRB2	RASSF2	FCGR2A	PTPRE	LCP1	ZFP36

Notes: The hub gene was calculated by cytoHubba 1; parameters 2; maximal clique centrality 3; density of maximum neighborhood component 4; maximum neighborhood component 5; edge percolated component 6; clustering coefficient 7.

## Discussion

### Main Goal for This Study

The aim of this study was to find the candidate genes by WGCNA. It could provide insights into the biology of early lung cancer and find the diagnostic biomarker by detecting the gene expression of nasal epithelia, which could make up for the shortage in postoperative pathological diagnosis and guide the clinical therapy. WGCNA has been used to not only construct gene networks and detect modules but also identify hub genes and select significant genes as biomarkers based on gene correlations. Module detection in WGCNA is used as a knowledge-independent process. However, empirical judgment and functional annotation would be more accurate, followed by the selection of a threshold for culling the network (Letovsky, 1987; Liu et al., 2015). WGCNA is considered a better prediction for hub genes when it comes to the biological process than the regression statical methods. Therefore, the construction of mutants will also help to detect the hub genes for prediction of lung cancer and to understand the role of specific genes in pathogenesis, which was overlooked in early lung cancer (Subudhi et al., 2015).

### Technology and Method of WGCNA

WGCNA was applied to investigate 3,600 genes downloaded from a dataset at NCBI. First, the data were performed to obtain the gene expression consensus modules of nasal epithelia, module eigengenes, clinical traits, and their relationships. Second, we constructed the status-specific modules of lung cancer. Third, we identified the hub genes in brown and blue modules through cytoHubba in Cytoscape and detected the related genes in 12 algorithms. Lastly, we performed the gene enrichment analysis on GO and pathway terms.

### New Results

WGCNA was used to investigate 3,600 genes downloaded from a dataset at NCBI. We obtained evidence about the changes of the hub gene expression in the feature gene module. The expressions of EMR3, NCF1, CSF2RB, DYSF, TLR8, and HCK in the lung cancer group were significantly different from those of the control group. The most significant difference in gene expression is EMR3, followed by NCF1, CSF2RB, DYSF, TLR8, and HCK.

### About EMR3

EMR3 is one of the members of the epidermal growth factor 7 transmembrane protein family (EGF-TM7), which includes CD97, EMR1, EMR2, and EMR4 and is expressed in the immune system cells. Until now, its functions are unclear yet, as well as the ligand and downstream signal (Stacey et al., 2001). Some research studies found that EMR3 is mainly expressed in mature granulocytes, and other members from the EGF-TM7 family may mediate the cell migration and leukocyte migration (Matmati et al., 2007; Yona et al., 2008a; Yona et al., 2008b). Ari and Kane found that EMR3 is expressed in glioblastoma cells and can mediate cell migration and invasion. It has the highest level of neutrophils, monocytes, and macrophages in the peripheral blood of Crohn’s patients (Kane et al., 2010).

### About NCF1

NCF1 is a major component of the nicotinamide adenine dinucleotide oxidase system; it can regulate the production of reactive oxygen species (ROS). NCF1 deficiency will lead to the reduction of ROS, which is associated with immune disorders (Bastos et al., 1995). NCF1-knock-out mice have increased leukocyte infiltration and morphological changes in the colonic mucosa, indicating that the absence of the NCF1 gene could aggravate colitis (El Naschie, 2004). In contrast, the upregulation of NCF1 gene expression might cause diminished or deficient ROS production that is detrimental to human health.

### About CSF2RB and Others

CSF2RB is the common beta chain of the high-affinity receptor complexes for ligands of IL-3, IL-5E, and CSF. Research studies found that mutation of CSF2RA or CSF2RB can cause hereditary pulmonary alveolar proteinosis (PAP) (Takaki et al., 2016), and CSF2RB is a risk factor for schizophrenia and depression in the Han population of Chinese and a potential oncogene that can be targeted by several miRNAs for undergoing cell apoptosis (Chen et al., 2011). The DYSF gene is a 220-kD protein, which plays a major role in the regulation of plasma membrane repair. Fusion of DYSF with the ALK gene has been found to be associated with advanced lung cancer. As a single-stranded RNA sensor, the activation of TLR8 can also promote the survival and chemoresistance of lung cancer cells. The HCK gene belongs to the Src family of tyrosine kinase, which is mainly involved in the regulation of polymorphonuclear leukocytes. A recent study showed that in the Bai nationality of China, the polymorphism of the introns of the HCK gene is associated with lung function and airway abnormality (Espinoza-Fonseca, 2016).

### Experimental Verification

Studies have proved that the existence of injury in the bronchial airway results in gene expression alterations in patients with lung cancer, and the airway epithelial injury associated with lung cancer extends to the nasal epithelium (Shannon et al., 2003). In the previous study, the downregulated genes CASP10 and CD177 and the upregulated genes BAK1, ST14, CD82, and MUC4 were detected as biomarkers for lung cancer by the joint sparse regression model (Loxham and Davies, 2017). Our study has detected some hub genes from gene expression of the nasal epithelium of early lung cancer by WGCNA. The most significant difference in gene expression was shown by EMR3, followed by NCF1, CSF2RB, DYSF, and so on. The results of qRT-PCR are in accordance with those of microarray analysis (Qureshi, 2018).

### Clinical Application

This study may provide an additional proof for detecting early lung cancer by observing gene expression of the nasal epithelium, which indicates a great potential for clinical application (Lobato and O'Sullivan, 2018). The biomarker of nasal epithelium would be used as a reference for patients with small nodules at low risk of malignancy, which can be managed by CT screening (Petty, 2001; Cottin and Cordier, 2016). However, this study still has some limitations. It lacks further studies on the relationship between gene expression and pathological typing of lung cancer (Tang et al., 2018), so large-scale samples must be collected to have a better analysis in the future.

## Data Availability

The original contributions presented in the study are included in the article/Supplementary Materials; further inquiries can be directed to the corresponding author.

## Abbreviations

DEGs, differentially expressed genes; GEO, Gene Expression Omnibus; GO, Gene Ontology; GS, gene significance; HRCT, high-resolution CT; KEGG, Kyoto Encyclopedia of Genes and Genomes; MM, module membership; MCC, modified Cam Clay; RMA, robust multi-array average; SYK, spleen tyrosine kinase; SCLC, small cell lung cancer; WGCNA, weighted gene co-expression network analysis.
